# Meteorological Register for August, 1817

**Published:** 1817-10

**Authors:** John Bailey

**Affiliations:** Surgeon, &c.


### Meteorological Register for August, 1817,

*Kept at Harwich, in Essex,*

By JOHN BAILEY, Surgeon, &c.

| Moon's Age. | | Atmospherical Preffure. Morn. | Ev. | Temp. | Wind. | Remarks. | General Statement of Difeafes. |
|---|---|---|---|---|---|---|---|
| | 1 | 29 7 | 29 7 | 66 | SW | Rain | |
| | 2 | 7 | 7 | 69 | S | | |
| | 3 | 6½ | 6½ | 63 | W | ditto. Windy | |
| | 4 | 6½ | 6½ | 66 | W | Rain | |
| ☾ | 5 | 8 | 8 | 68 | E | Fine and clear | |
| | 6 | 30 | 30 | 68 | SW | | |
| | 7 | 29 9 | 29 7 | 69 | S | Rain | Rubeola |
| | 8 | 6 | 6 | 64 | SW | Cloudy & windy | |
| | 9 | 6½ | 7 | 66 | SW | Rain. Windy | |
| | 10 | 8 | 8 | 64 | W | ditto ditto | Cholera |
| | 11 | 8 | 5 | 65 | SW | ditto ditto | |
| ○ | 12 | 4 | 4 | 67 | SW | ditto ditto | |
| | 13 | 2½ | 6 | 65 | SW | ditto ditto | Rheumatismus Acutus |
| | 14 | 6 | 6 | 69 | SW | ditto. Gale of wind | |
| | 15 | 6½ | 6½ | 69 | S | ditto ditto | |
| | 16 | 8 | 5 | 66 | SW | ditto | |
| | 17 | 6 | 7 | 60 | NW | Violent rain | |
| | 18 | 8 | 8 | 65 | SW | Rain | |
| ☽ | 19 | 7 | 7 | 66 | SW | Much rain | |
| | 20 | 5½ | 6 | 66 | SW | Rain | |
| | 21 | 6 | 9 | 64 | W | ditto | |
| | 22 | 9 | 30 | 63 | NW | ditto | |
| | 23 | 29 9 | 29 9 | 62 | SE | Fine and clear | |
| | 24 | 8 | 8 | 62 | S | Dull and cloudy | |
| | 25 | 2 | 1½ | 60 | SW | Rain | |
| ● | 26 | 1½ | 1½ | 64 | SW | Much rain | |
| | 27 | 1 | 4 | 65 | S | Rain | |
| | 28 | 5½ | 6½ | 66 | W | Rain | |
| | 29 | 5½ | 7 | 65 | W | Much r. tempest | |
| | 30 | 7½ | 8 | 67 | W | Fine | |

Towards the latter end of the present month an isolated case of Measles occurred in a large family; mild in its attack and progress, but so well characterized as to speak for itself in unambiguous terms. The other parts of the family, on closing this Report, had not been infected.

Cholera has been very general, but not so violent as on former visitations.

The low state of the Thermometer, accompanied by continual rain, has set back convalescents in acute Rheumatism. Three patients, who laboured for many weeks under this tiresome complaint, imagined themselves well, and got out to enjoy a little fresh air, but were attacked again, and run through a series of suffering more violent than at first.

